# Acute Profound Hypotension Following Intraoperative Plasma Transfusion—A Case Series

**DOI:** 10.1111/aas.70365

**Published:** 2026-10-25

**Authors:** Tommy Batz, Johan Nordström, Daniel Hertzberg, Vendela Brismar, Jessica Kåhlin, Magnus Hedberg, Erik Von Oelreich, Malin Jonsson Fagerlund

**Affiliations:** ^1^ Perioperative Medicine and Intensive Care Karolinska University Hospital Stockholm Sweden; ^2^ Section for Anaesthesiology and Intensive Care Medicine, Department of Physiology and Pharmacology Karolinska Institutet Stockholm Sweden; ^3^ Department of Clinical Immunology and Transfusion Medicine Karolinska University Hospital Stockholm Sweden

## Abstract

**Background:**

Blood transfusions can be lifesaving but carry risks of serious adverse reactions, including the less recognized hypotensive transfusion reaction, characterized by hypotension that may be difficult to distinguish from other transfusion reactions or haemorrhage‐related shock. At the Karolinska University Hospital, an increased incidence of perioperative hypotensive transfusion reactions without typical allergic features has been observed during recent years. The aim of this study was to identify and describe common clinical characteristics among patients who experienced perioperative transfusion reactions.

**Methods:**

This case series utilized data from a local quality registry at the Karolinska University Hospital, Sweden, covering the period from June 2024 to June 2025. Data collected included patient demographics, relevant clinical parameters and summaries of each reaction. Descriptive statistics were applied to identify potential common traits.

**Results:**

Seventeen patients with perioperative transfusion reactions were included. All but one developed hypotension and in 11 cases hypotension was the sole manifestation. None of these 11 showed elevated serum tryptase levels. All patients except one received plasma via a Belmont Rapid Infuser RI‐2 and seven patients also received packed red blood cells. In 11 cases, the blood pressure drop occurred immediately after plasma infusion. During this period, the Belmont Rapid Infuser was withdrawn after 7 months. No reactions were reported in the 4 months following withdrawal. However, reactions recurred following subsequent device use.

**Conclusion:**

Most transfusion reactions were characterized by profound and immediate hypotension following plasma transfusion, often presenting as isolated hypotension without biochemical or clinical evidence consistent with IgE‐mediated anaphylaxis. A temporal association with the use of the Belmont Rapid Infuser RI‐2 was observed. These findings should be regarded as hypothesis‐generating, warranting further prospective mechanistic and safety studies.

**Editorial Comment:**

Hypotensive transfusion reactions are uncommon but clinically important, and may be mistaken for bleeding or anaphylaxis. This case series describes 17 perioperative cases, mainly presenting as abrupt isolated hypotension after plasma administration. The absence of device‐use denominators, signs of anaphylaxis in most patients and the incomplete, delayed calcium measurements mean that other mechanisms cannot be separated from a device‐related effect. The main message for practice is therefore active vigilance when using a rapid plasma infusion device.

## 1 Introduction

Blood transfusions can be lifesaving but also carry the risk of serious adverse reactions, including transfusion‐associated circulatory overload (TACO), transfusion‐related acute lung injury (TRALI), transfusion‐related sepsis, acute haemolytic transfusion reaction and anaphylaxis [1]. Hypotension is a common sign across several of these reactions but may also be part of the clinical presentation necessitating the transfusion, particularly in the context of haemorrhage.

A less commonly recognized entity is the hypotensive transfusion reaction (HyTR), also referred to as acute hypotensive transfusion reaction (AHTR). Hypotensive transfusion reaction is defined as a sudden decrease in systolic blood pressure (SBP) of ≥ 30 mmHg and an absolute SBP of ≤ 80 mmHg occurring during or within 1 h after transfusion, in the absence of another identifiable cause [2].

Clinical guidelines recommend stopping the transfusion in case of hypotension, as HyTR often resolves upon cessation of transfusion. However, when hypotension coincides with active bleeding, it is frequently attributed to hypovolemia and transfusion may continue uninterrupted. This overlap complicates recognition and likely contributes to the aggravation and underreporting of HyTR.

Additionally, blood warmers have emerged as a potential contributing factor to transfusion reactions. Over a 4‐year period, two centres jointly reported 15 cases of HyTR associated with transfusion using the Belmont Rapid Infuser RI‐2 (Belmont Medical Technologies, Billerica, United States of America), although the underlying aetiology remains unclear [3].

While several case reports have described HyTR, its pathophysiology is poorly understood. Some reports suggest that angiotensin‐converting enzyme (ACE) inhibitors may play a role, as these medications inhibit bradykinin degradation, leading to its accumulation and a subsequent vasodilatory effect [4, 5, 6, 7, 8, 9, 10].

At the Karolinska University Hospital in Solna, Sweden, an increased incidence of transfusion reactions has been observed during recent years, most occurring without accompanying signs of allergic response. This concern arose from a quality improvement project that identified a high rate of suspected transfusion reactions during elective abdominal aortic surgery. Approximately 7% of 72 cases were classified as severe transfusion reactions, typically occurring shortly after the initiation of plasma transfusion during a hemodynamically stable phase and before aortic clamp release (unpublished data). These findings prompted the initiation of a systematic data collection of patients with these reactions to further investigate the phenomenon.

## 2 Methods

A local registry for systematic data collection on perioperative transfusion reactions was established at the Karolinska University Hospital, Solna. Adult patients undergoing non‐cardiac surgery were eligible for inclusion and were identified through standardized reporting forms. All operating room staff were informed that a specific transfusion reaction protocol should be completed in addition to the standard adverse event report whenever a transfusion reaction was identified (Figure 1). Patients who underwent surgery between June 26, 2024 and June 5, 2025, were included in the present study. Ethical permission was obtained from the Swedish Ethical Review Authority (Dnr 2025‐04895‐01).

**FIGURE 1:**
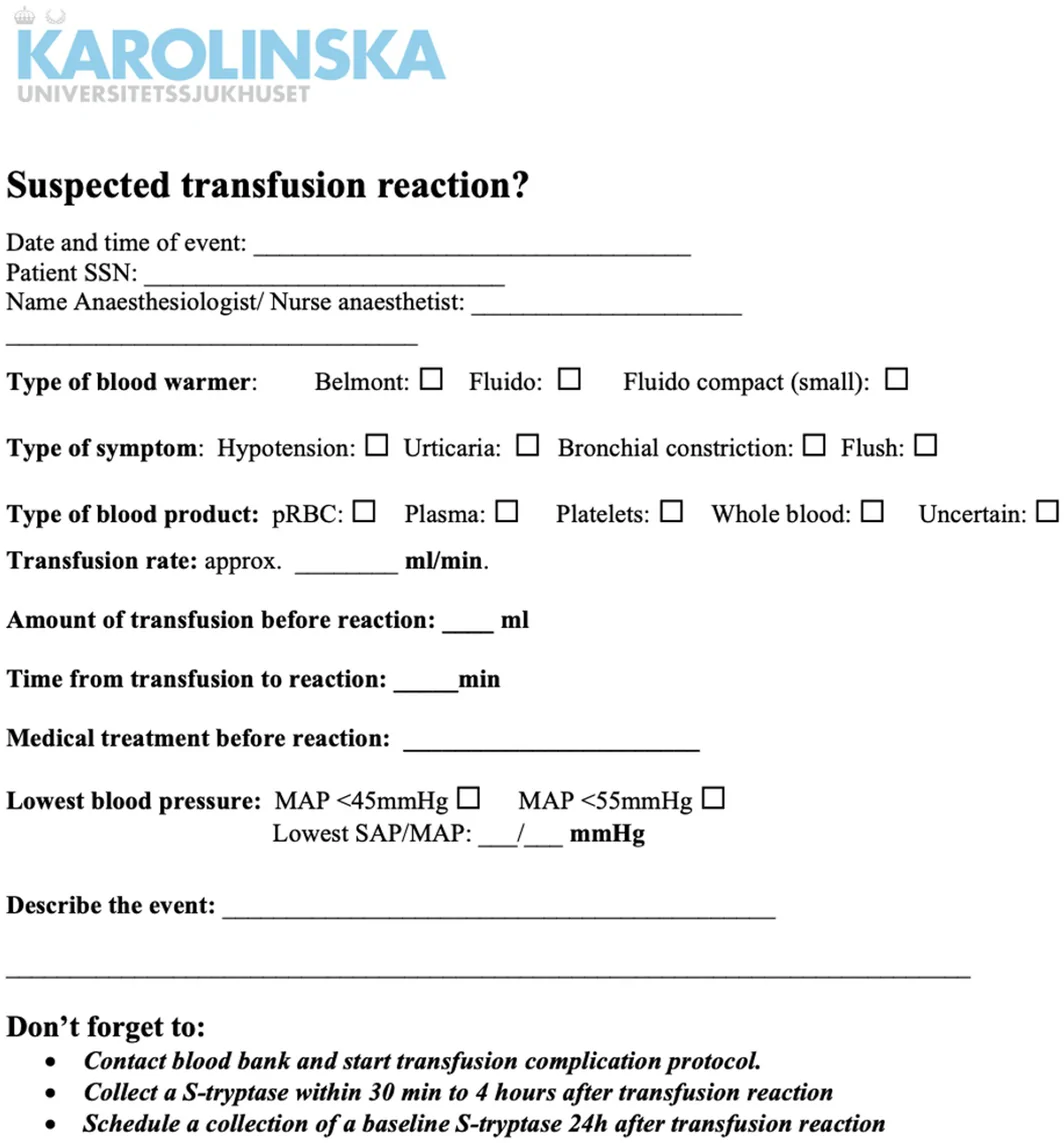
Transfusion reaction protocol (translated from Swedish). Filled in by anaesthesiologist after a patient had a suspected transfusion reaction.

Patients with a transfusion reaction were managed according to institutional routines, which included appropriate pharmacological interventions, return of the implicated blood product to the blood bank, collection of relevant blood samples and measurements of serum tryptase. As recommended in current guidelines, serum tryptase samples were collected 30 min after symptom onset and again approximately 24 h after complete resolution of symptoms to establish a baseline value. An increase in serum tryptase greater than (baseline × 1.2) + 2 μg/L was interpreted as elevated [11]. Analyses of tryptase were conducted at the Karolinska University Hospital laboratory.

The transfusion reaction protocol included detailed documentation of each transfusion event, capturing information on the use of a blood warmer, characteristics of the reaction, the type and rate of blood product administration, drugs given prior to the reaction and the lowest recorded blood pressure. A brief narrative description of each reaction was also included. Continuous high‐resolution haemodynamic data were available for one patient only. If any checkbox in the reporting form (e.g., for urticaria) was left unmarked, it was interpreted as absence of the symptom rather than as missing data. Transfusion of blood products was administered at the discretion of the treating clinician, based on the clinical context, including bleeding status, suspected coagulopathy and use within massive transfusion protocols. No study‐specific or protocolised criteria were applied. Indications for transfusion were not recorded in a structured format.

Additional information was collected from the anaesthesia records and medical report, such as patient demographics, comorbidities, type of surgery, vasoactive medication use, serum tryptase levels, blood type and results of serological testing.

Descriptive statistics were used to summarize patient demographics, laboratory values and event characteristics. Continuous variables were reported as medians with interquartile ranges. Categorical variables were presented as counts and percentages. All statistical analyses were performed using IBM SPSS Statistics, version 31 (IBM Corp., Armonk, NY, USA).

## 3 Results

A total of 17 patients with perioperative transfusion reactions were reported between June 26, 2024 and June 5, 2025. The mean patient age was 62 years (range 33–81 years) and 59% were male (Table 1). Each case was reported by a different anaesthesiologist, except for one clinician who reported two separate transfusion reactions. Individual patient and clinical characteristics are presented in Table 2.

**TABLE 1:** Summary of patient and clinical characteristics.

	Total cohort
Sex, male	10 (59)
Age (years)	64 (58–72)
ASA‐classification	
I	3 (18)
II	4 (24)
III	8 (47)
IV	2 (12)
ABO Rh group	
O pos	5 (29)
O neg	3 (18)
A pos	6 (35)
A neg	1 (6)
B pos	2 (12)
Acute surgery	5 (29)
Elective surgery	12 (71)
Type of surgery	
Abdominal	6 (35)
Abdominal aortic surgery	2 (12)
Protatectomy	3 (18)
Other^a^	6 (35)
Lowest MAP	
< 55 mmHg	3 (18)
< 45 mmHg	13 (76)
*Missing values*	1
Symptoms	
Hypotension	16 (94)
Urticaria	5 (29)
Bronchial Constriction	1 (6)
Rashes	1 (6)
Serum tryptase taken	
Shortly after transfusion reaction	11 (65)
Baseline	6 (35)
Elevated serum tryptase^b^	2 (12)
Ionised calcium measured	
Within 1 h of suspected reaction	13 (76)
< 1.1 mmol/L within 1 h of suspected reaction	11 (65)
< 1.0 mmol/L within 1 h of suspected reaction	6 (35)
< 0.9 mmol/L within 1 h of suspected reaction	1 (6)
Transfusion rate (mL/min) *Missing values*	100 (45–200)
Total blood loss at end of surgery (ml)	3400 (2254–7200)
Type of blood product^c^	
Plasma	17 (100)
pRBC	7 (41)
Platelets concentrates	1 (6)
Adrenaline	8 (47)
*Missing values*	1
Noradrenaline	13 (76)
*Missing values*	4
ACE inhibitors	4 (24)
PONV prophylaxis with steroids prior to transfusion reaction	13 (76)
*Missing values*	3

*Note:* Data are presented as *n* (%) for categorical variables and median (Q1–Q3) for continuous variables.

^a^ Other types of surgery included coiling of gastroduodenal artery, gastroscopy, hysterectomy, surgical removal of pituitary adenoma, surgical removal of spinal tumour and vascular neurosurgery.

^b^ Elevated serum tryptase was a transfusion reaction associated sample greater than baseline × 1.2 + 2.

^c^ Type of blood product suspected of causing transfusion reaction.

**TABLE 2:** Patient and clinical characteristics for individual cases.

Case #	Gender	Age (years)	ASA	Lowest MAP (mmHg)	ACE‐inhibitor treatment	PONV‐prophylactic corticosteroids prior to transfusion	Other symptoms than hypotension	Serum tryptase/baseline tryptase (μg/L)	ABO and Rh group	Transfusion rate (ml/min)	Noradrenaline infusion prior to reaction (μg/kg/min)	Adrenaline (mg)	Total bleeding (ml)	Type of blood product	Acute or elective	Type of surgery	Lowest ionised calcium measured within 1 h of reaction (mmol/L)
1	Female	81	II	< 45	No	Yes	Rashes	X/X	O Pos	X	0	0	0	Plasma	Acute	Coiling of gastroduodenal artery	0.95
2	Female	58	I	< 45	No	X	—	5.1/X	O Pos	30	X	0.1	200	Plasma	Acute	Laparotomy	1.25
3	Male	74	III	< 45	Yes	Yes	—	X/X	A Pos	< 30	0.03	0	2900	Plasma	Elective	AAA	1.08
4	Male	63	IV	< 45	No	X	Urticaria, respiratory issue	X/X	A Neg	X	X	Yes, but unknown dose	3000	Plasma	Acute	Gastroscopy	X
5	Male	66	II	< 45	Yes	Yes	Urticaria	X/X	O Neg	200	0.14	0.04	11,900	Plasma + pRBC	Elective	Laparotomy	1.08
6	Male	64	III	< 45	No	Yes	Urticaria	14/6	O Pos	200	0.08	0.05	7200	Plasma + pRBC	Elective	Laparotomy	X
7	Female	59	I	< 55	No	Yes	—	X/X	B Pos	50	0.06	0	2254	Plasma	Elective	Hysterectomy	0.91
8	Female	81	III	< 45	Yes	Yes	—	X/X	O Pos	100	0.08	0.01	15,000	Plasma	Elective	Robot assis. Proctectomy	1.1
9	Male	72	III	< 55	No	Yes	—	13/10	A Pos	30	0.08	0	1590	Plasma	Elective	AAA	1.07
10	Male	65	II	< 45	No	Yes	—	4.2/2.2	A Pos	100	0.06	0.025	10,900	Plasma + pRBC	Elective	Laparotomy	0.91
11	Male	60	II	< 45	No	Yes	—	2.7/3	O Neg	100	0.09	0	24,100	Plasma	Elective	Proctectomy	0.96
12	Female	53	III	< 55	No	Yes	—	3.2/X	A Pos	200	0.04	0	3785	Plasma + pRBC	Acute	Surgical removal of spinal tumour	1.09
13	Male	52	IV	< 45	No	X	—	11/9	A Pos	500	Yes, but no value	0	1150	Plasma	Acute	Vascular neurosurgery	X
14	Female	44	III	X	No	Yes	Only urticaria^a^	19/X	B Pos	X	X	0	5300	Plasma + pRBC	Elective	Proctectomy	X
15	Female	32	I	< 45	No	No	—	4.8/X	A pos	X	0	0	2500	Plasma + pRBC	Acute	Laparotomy after c‐section	0.95
16	Male	80	III	< 45	No	Yes	Urticaria	24/7	O Neg	X	0.07	1.4	3400	Plasma + pRBC	Elective	Transsphenoidal surgery	0.76
17	Male	69	III	< 45	Yes	Yes	—	4.5/X	O Pos	150	0.06	0.06	6436	Plasma	Elective	Laparotomy	1.04

*Note:* X is for missing data (information not recorded or not available) and 0 is when it's confirmed that it was not administered (confirmed absence/non‐administration).

^a^ Case #14 did not have hypotension as a symptom, only urticaria.

A mean arterial pressure (MAP) below 55 mmHg was recorded in 16 patients, of whom 13 had a MAP lower than 45 mmHg (Table 1, Figure 2). The single patient who did not develop hypotension exhibited only localized urticaria. Five patients showed additional manifestations such as rash or urticaria and in one of these cases, the reaction was accompanied by bronchoconstriction. Postoperative intensive care unit admission was required for one patient who developed airway swelling related to the transfusion reaction.

**FIGURE 2:**
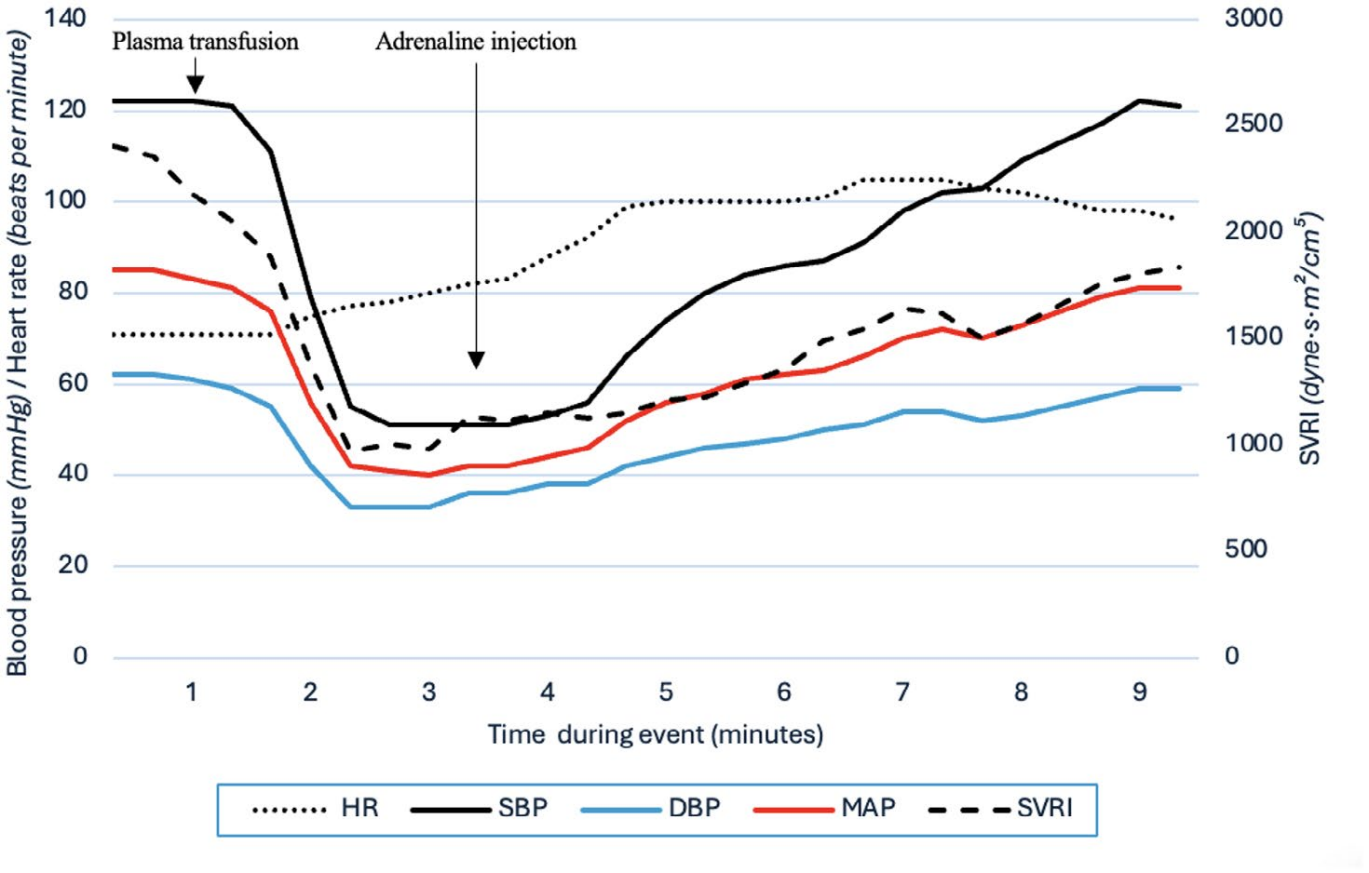
Example of hemodynamic changes during a transient hypotensive event. Systolic blood pressure (SBP), diastolic blood pressure (DBP), mean arterial pressure (MAP), heart rate (HR) and systemic vascular resistance index (SVRI) were measured over a 9‐min period with invasive blood pressure monitoring. SVRI was estimated by FloTrac device (Edwards Lifesciences Corporation, United states of America, Irvine). Plasma transfusion (arrow) was followed by a rapid decrease in blood pressures and accompanied by a compensatory increase in HR. Adrenaline injection (arrow) was associated with gradual recovery of blood pressures and SVRI.

All patients received plasma transfusion and seven patients additionally received packed red blood cells. Platelet concentrates were administered in one case. In 11 cases, the responsible anaesthesiologist noted that the blood pressure drop occurred suddenly and immediately after initiation of plasma infusion. Ten patients experienced reactions during elective surgery and the remaining five patients during emergency procedures. The median total blood loss during surgery or intervention was 3400 (IQR 2254‐7200) mL. However, the exact amount of blood loss and transfused blood product volume at the time of each transfusion reaction could not be retrospectively estimated. In many of these cases, a sudden unexplainable hypotension was observed shortly after start of plasma transfusion without simultaneously ongoing massive bleeding that could explain a significant drop in blood pressure. Invasive blood pressure monitoring was used in 16 of 17 patients; an example of haemodynamic trends during a reaction is shown in Figure 2.

All cases involved the use of the Belmont Rapid Infuser RI‐2 blood warmer except for one case in which the type of device was not documented. While using the Belmont Rapid Infuser, transfusion rates ranged from < 30 to 500 mL/min, with a median of 100 (IQR 45–200) mL/min.

Noradrenaline was administered prior to the reaction in 13 patients, at infusion rates ranging from 0.03 to 0.14 μg/kg/min; three of these patients did not receive any additional vasopressors. Adrenaline was used to treat the transfusion reaction in eight cases, with doses ranging from 0.005 to 0.1 mg; however, one patient who required cardiopulmonary resuscitation received a total cumulative dose of 1.4 mg. Ephedrine or phenylephrine was administered to five patients to treat the transfusion reaction. Four patients were on ACE inhibitors, although none had taken the drug on the day of surgery.

Serum tryptase samples were obtained from 11 of 17 patients in total. Both baseline and reaction‐associated tryptase levels were available for six of these patients. Among the six patients who presented with urticaria or rashes, serum tryptase samples were obtained in three; two of those also had baseline values and fulfilled the criteria for an IgE‐mediated allergic reaction, with delta serum tryptase levels of 8 and 17 μg/L, respectively. The third patient, for whom no baseline sample was available, had a reaction‐associated tryptase level of 19 μg/L. Among the 11 patients who experienced hypotension as the sole symptom, no tryptase was collected in three cases and four had low reaction‐associated tryptase levels without baseline samples. The remaining four patients had non‐elevated tryptase levels. Notably, one patient had a baseline tryptase level of 10 μg/L and a reaction‐associated level of 13 μg/L; however, the samples were collected only 10 h apart.

Ionised calcium within 1 h after the reaction was documented in 13 of 17 patients. One patient had an ionised calcium level below 0.9 mmol/L within 1 h of suspected reaction. No arrhythmias related to hypocalcaemia were observed. According to our departmental protocol, hypocalcaemia was treated with calcium gluconate to achieve an ionised calcium concentration above 1.0 mmol/L.

A summary of the data after 7 months revealed that all cases up to that point had involved transfusion of plasma in the Belmont device. Following this finding, a directive from the department was issued recommending discontinuation of the Belmont device. Instead, an alternative system was introduced when available. In situations without access to this alternative system, pRBC continued to be transfused using the Belmont, while plasma was administered separately using manual overpressure. When manual infusion proved inadequate to maintain sufficient flow rate, the Belmont device was occasionally used also for plasma transfusion. After discontinuing the use of the Belmont device in favour of an alternative warming system, the rate of reactions decreased. During the following 5 months, only two transfusion reactions were reported, both involving plasma administered through the Belmont device to achieve adequate flow rates in critical situations where manual compression proved insufficient. Throughout the inclusion period, the local blood bank maintained its usual blood product handling practices without alteration.

## 4 Discussion

This case series describes 16 patients with profound hypotension temporally associated with plasma transfusion during non‐cardiac surgery at a large tertiary referral centre. Notably, in most of the hypotensive patients, there was no clinical or biochemical evidence for IgE‐mediated anaphylaxis. All transfusions were administered using the Belmont RI‐2 system. After implementation of an alternative warming system, no further reactions were observed until the Belmont blood warmer was reintroduced for high‐flow massive transfusion in two cases. While these observations suggest a temporal association, they should be interpreted with caution, as causal relationships cannot be established and multiple contributing factors may be involved.

When compared with national transfusion vigilance data, in which only five plasma transfusion reactions were reported in Sweden in 2018 (four anaphylactic and one severe allergic reaction) [12], the frequency observed in this case series appears unexpectedly high. One possible explanation is an increased awareness effect, whereby heightened attention to a phenomenon leads to increased detection without a true change in incidence. However, the events were reported by multiple independent anaesthesiologists, reducing the likelihood of reporting bias or individual practice‐related effects. Although the number of cases is limited, these findings raise concern that acute HyTR may be underrecognized and underreported in the perioperative setting.

The repeated temporal association between plasma transfusion and profound hypotension observed with the Belmont Rapid Infuser RI‐2 in this study suggests a possible device‐related mechanism. Comparable events involving the same device have been reported previously, although not exclusively in connection with plasma transfusion [3, 13]. In contrast, a separate retrospective analysis identified platelets, cell salvaged blood, autologous blood and RBCs as the predominant triggers of hypotensive transfusion reactions [14].

In the present case series, however, all reactions were associated with plasma administration. Seven patients also received RBCs. In 11 cases the onset of hypotension occurred immediately following the initiation of plasma transfusion. None of the patients in this series underwent liver transplantation, whereas 87% of the cases described by Miller et al. occurred in that particular setting [3].

A key question is whether the observed hypotension could instead be explained by haemorrhage or relative hypovolemia. This is an inherent challenge in transfusion medicine, particularly in surgical patients with ongoing or potential bleeding. However, several features argue against simple hypovolemia as the primary mechanism in these cases. In most patients, hypotension occurred abruptly and immediately after initiation of plasma transfusion, often within seconds and at relatively low transfusion rates, in the absence of active haemorrhage. In many cases blood pressure normalized rapidly once transfusion was paused and vasopressor support was administered, without recurrence thereafter. The absence of progressive hemodynamic deterioration, which would typically be expected in haemorrhagic shock, further supports the interpretation that these were transfusion‐related reactions rather than the consequences of volume loss.

Serum tryptase measurement is the current standard of biomarkers for confirming mast cell activation in anaphylaxis [15]. However, the diagnostic sensitivity is variable and correlates with the severity and timing of sampling [16]. In this study, tryptase testing was inconsistent and in most patients with isolated hypotension, levels were not elevated. Although the absence of tryptase elevation cannot completely exclude anaphylaxis [15], particularly when sampling is delayed, the clinical features, namely the lack of cutaneous or respiratory symptoms and the limited need for adrenaline, argue against an IgE‐mediated mechanism. The pattern of abrupt, isolated hypotension resolving upon cessation of transfusion aligns more closely with previously described cases of HyTR than with the European Academy of Allergy and Clinical Immunology's (EAACI) guidelines for anaphylaxis [17].

It is noteworthy that administration of corticosteroids as prophylaxis for postoperative nausea and vomiting (PONV) prior to transfusion did not prevent these reactions. This finding is consistent with a prior study of non‐haemolytic transfusion reactions, suggesting that PONV prophylactic steroids are ineffective in preventing HyTR, although their influence on reaction severity remains uncertain [18].

The role of angiotensin‐converting enzyme (ACE) inhibition in HyTR has been well described, as ACE inhibition reduces bradykinin degradation and can predispose patients to exaggerated vasodilatory responses [4, 5, 6, 7, 8]. However, in our cohort, only four patients were on ACE inhibitors and none had taken the drug on the day of surgery. These findings suggest that while ACE inhibition can potentiate HyTR, it is unlikely to have been a major contributing factor in this series.

An important alternative mechanism that was not assessed in this study is citrate‐induced hypocalcaemia. Hypocalcaemia is a recognized cause of cardiovascular depression, reducing cardiac contractility and vasomotor tone and can present as acute hypotension [19]. Ionised calcium measurements were available within 1 h of the reaction in 13 of 17 patients. The majority of these had at one time‐point levels below 1.1 mmol/L, six below 1.0 mmol/L, but only one patient had an ionised calcium below 0.9 mmol/L. Importantly, ionised calcium measurements were not protocolised and were therefore not obtained at the exact time of the hypotensive event and may not reflect the value at the hypotensive event. Acute reductions in ionised calcium during citrate exposure can occur within minutes and may also normalize relatively rapidly due to citrate metabolism, calcium administration or paused transfusions. However, hypocalcaemia alone does not explain the apparent clustering of events with a specific transfusion device.

Other mechanisms such as complement activation and bradykinin generation, which can occur during blood product processing and warming, cannot be excluded and should be evaluated in future mechanistic investigations.

The clinical implications of these findings are relevant for transfusion practice and system safety. Firstly, HyTR should be considered in the differential diagnosis of sudden intraoperative hypotension during transfusion, even in the absence of allergic features. Secondly, the consistent association with one specific infusion device in this study highlights the need for vigilance and post‐market safety evaluation of transfusion equipment. Following the identification of these events, our institution discontinued the use of the Belmont device for plasma administration, introducing an alternative warming system. Continued surveillance is ongoing to determine whether this change reduces the incidence of hypotensive reactions.

This study has several limitations. The case reporting form was intentionally concise to encourage reporting compliance, which restricted data granularity. Detailed changes in vasoactive drug infusion rates and haemodynamic recovery could not be retrospectively reconstructed. In addition, the frequency of use of each blood warming device was not recorded, limiting the ability to quantify relative risk. The absence of denominators for device‐specific plasma transfusion rates prevents estimation of the true incidence or relative risk attributable to the Belmont device. Moreover, the sample size was small and derived from a single centre. Incomplete laboratory testing, particularly missing tryptase samples, further limits mechanistic conclusions. Despite these constraints, the structured data collection provides valuable descriptive information on an uncommon but clinically relevant transfusion complication. The specific plasma product was not systematically recorded in the dataset. However, in our institution, standard practice during the study period was to use fresh frozen plasma (FFP) from single donors rather than pooled solvent–detergent plasma.

This case series demonstrates that profound hypotension associated with plasma transfusion in the perioperative setting is unlikely to be explained by IgE‐mediated anaphylaxis. The absence of tryptase elevation in patients with isolated hypotension, the lack of consistent cutaneous or respiratory features and notably the limited use of adrenaline and the low doses administered argue strongly against this mechanism. The temporal association with a specific device should be regarded as a hypothesis‐generating observation. The available data do not allow determination of the underlying mechanism. We encourage other hospitals to report similar events to their local transfusion services and national haemovigilance programmes.

## Author Contributions

T.B., J.N. and D.H. identified the clinical phenomenon and contributed to the conception of the study. T.B., E.V.O. and M.J.F. established the registry, coordinated data collection, performed data analyses and drafted the manuscript. All authors contributed to the study design, interpretation of data and critical revision of the manuscript. M.J.F. supervised the study. All authors reviewed and approved the final manuscript.

## Funding

This study was supported by Karolinska Institutet and Region Stockholm.

## Conflicts of Interest

The authors declare no conflicts of interest.

## Data Availability

The data that support the findings of this study are available from the corresponding author upon reasonable request.
